# Baseline Factors Associated with Pain Intensity, Pain Catastrophizing, and Pain Interference in Intensive Interdisciplinary Pain Treatment for Youth

**DOI:** 10.3390/children10071229

**Published:** 2023-07-15

**Authors:** Rob D. Long, Andrew Walker, Si Chen Pan, Jillian Vinall Miller, Laura Rayner, Joanne Vallely, Nivez Rasic

**Affiliations:** 1 Cumming School of Medicine, University of Calgary, Calgary, AB T2N 4N1, Canada; 2 Anesthesiology, Perioperative & Pain Medicine, University of Calgary, Calgary, AB T2N 4N1, Canada; 3 Vi Riddell Children’s Pain & Rehabilitation Centre, Alberta Children’s Hospital, Calgary, AB T3B 6A8, Canada; 4 Child Brain & Mental Health Program, Alberta Children’s Hospital Research Institute, Calgary, AB T2N 4N1, Canada; 5 Owerko Centre, Alberta Children’s Hospital Research Institute, Calgary, AB T2N 4N1, Canada; 6 Brain & Behaviour Team, Hotchkiss Brain Institute, Calgary, AB T2N 4N1, Canada; 7 Mathison Centre for Mental Health Research & Education, Hotchkiss Brain Institute, Calgary, AB T2N 4N1, Canada; 8 Department of Psychology, University of Calgary, Calgary, AB T2N 1N4, Canada; 9 O’Brien Institute for Public Health, University of Calgary, Calgary, AB T2N 4Z6, Canada

**Keywords:** chronic pain, interdisciplinary pain treatment, catastrophizing, quality of life, anxiety, children, adolescents, parent-child, longitudinal research

## Abstract

**Background:** More could be known about baseline factors related to desirable Intensive Interdisciplinary Pain Treatment (IIPT) outcomes. This study examined how baseline characteristics (age, gender, child pain catastrophizing (PCS-C), pain interference, pain intensity, anxiety, depression, paediatric health-related quality of life (PedsQL^TM^), and parent catastrophizing (PCS-P)) were associated with discharge and 3-month follow-up scores of PCS-C, pain intensity, and pain interference. **Methods:** PCS-C, pain intensity, and pain interference T-scores were acquired in 45 IIPT patients aged 12–18 at intake (baseline), discharge, and 3-month follow-up. Using available and imputed data, linear mixed models were developed to explore associations between PCS-C, pain intensity, and pain interference aggregated scores at discharge and follow-up with baseline demographics and a priori selected baseline measures of pain, depression, anxiety, and PCS-C/P. **Results:** PCS-C and pain interference scores decreased over time compared to baseline. Pain intensity did not change significantly. Baseline PCS-C, pain interference, anxiety, depression, and PedsQL^TM^ were associated with discharge/follow-up PCS-C (available and imputed data) and pain interference scores (available data). Only baseline pain intensity was significantly associated with itself at discharge/follow-up. **Conclusions**: Participants who completed the IIPT program presented with reduced PCS-C and pain interference over time. Interventions that target pre-treatment anxiety and depression may optimize IIPT outcomes.

## 1. Introduction

Chronic pain is experienced by 1 in 5 children and youth, generally affecting girls more than boys [1]. It negatively impacts physical, emotional, and social functioning, and the wellbeing of children and their families [2,3]. Chronic pain is one of the leading causes of human suffering and disability [4]. Evidence-based therapies can include psychological interventions, physical therapies, and medications or medical interventions, delivered in a variety of formats for variable periods of time [5]. For youth with complex, treatment-resistant chronic pain associated with poor functioning, Intensive Interdisciplinary Pain Treatment (IIPT) is often the treatment of choice [6], having been shown to improve pain intensity, disability, and psychosocial outcomes in this population [6,7,8].

IIPT programs aim to help patients self-manage pain, improve function, and resume participation in meaningful activities, rather than eliminate pain symptoms altogether [9]. They involve at least three professional groups, including physicians, psychologists, and physical therapists working together with shared treatment goals over an average of 2–6 weeks [10,11]. Programs also work with parents to equip them with skills and resources to care for a child with chronic pain [5]. A growing body of research demonstrates that IIPT is effective in improving outcomes. Recent systematic reviews show significant reductions in mean pain intensity and disability at discharge and 2–24 month follow-up [6,7]. Longer-term studies suggest that these improvements may be retained for several years after treatment [12,13,14]. Parents experience direct benefits from IIPT as well, with improvements in their own depression and anxiety symptoms, pain catastrophizing, and parenting behaviour [15]. There is also an economic benefit as IIPT programs reduce costs to the healthcare system, including less medication use and reduced hospital stays [16,17]. Psychosocial outcomes in youth have been shown to improve, including decreases in pain catastrophizing, depressive symptoms, and emotional distress, as well as an improvement in quality of life [8].

Despite promising reductions in a range of important outcomes, not everyone improves after IIPT. A recent randomized control trial of IIPT in youth showed that only 65% of patients demonstrated improvement in pain intensity, disability, and school absence at 12 month follow-up [18]. Moreover, a longitudinal observational study found that improvements in pain-related, psychological, and economic variables were sustained in 60% of patients at 4 years [13]. Both studies called for investigation into pretreatment factors such as gender, mood, emotion, and pain characteristics that could be used to better predict treatment outcomes [13,18].

Recently, Simons et al. performed a trajectory analysis to determine who responds to IIPT treatment using pain intensity as one of their primary outcomes [14]. They categorized patients into groups of responders or non-responders in terms of clinically relevant changes in pain intensity and functional disability, and then looked at baseline characteristics associated with these groups. For pain intensity, older age, inability to take a ‘self-management’ approach, and anxiety were associated with non-responding. Interestingly, having more social difficulties before treatment was associated with an improved response. The authors suggest that this may be due to the relative improvement in wellbeing that comes with a transition to a more understanding social milieu. Of note, parental pain catastrophizing was not associated with pain intensity trajectories in this study. This research provides valuable insight into factors that may influence response to treatment in terms of pain intensity.

Pain intensity contributes significantly to quality of life [19]. The relationship between pain and depression is strongly influenced by pain interference, and there is an indirect effect of pain interference on the relation between pain and functional disability [20]. Paediatric chronic pain patients also express interest in including pain intensity as an outcome for IIPT treatment [21,22]. Thus, pain intensity is a desirable measure for many IIPT programs to include in data collection.

However, the relative importance of improving psychosocial outcomes in IIPT compared to improving pain intensity has become clearer. One outcome of particular importance is pain catastrophizing. Pain catastrophizing can be understood as a set of anxiety-driven, heightened emotional responses to, and predictions about, pain that lead to difficulty coping with a painful experience [23]. It is a key concept within the Fear Avoidance Model of pain (FAM), which postulates that an attitude towards pain that increases a person’s avoidant behaviours contributes to the harmful impacts of chronic pain and delays recovery [24]. In a systematic review of studies on pain catastrophizing, it was found to be more strongly correlated to functional disability than pain intensity [25]. In a meta-analysis on the same topic, pain catastrophizing also had a strong relationship with anxiety, depression, and quality of life [26]. Parent pain catastrophizing has also been associated with child fear avoidance processes, such as child pain catastrophizing [27]. These findings suggest that targeting pain catastrophizing could have far reaching benefits. Pain catastrophizing is a desirable outcome measure in IIPT given its close relationship with disability and its ability to be targeted by focused psychosocial interventions. In this setting, it may be more useful to measure pain catastrophizing than pain intensity.

Another important outcome in IIPT is pain interference. Pain interference measures the degree to which pain prevents patients from achieving age-appropriate physical, psychological, and social functioning [28]. Higher pain interference scores can be understood to represent worse disease severity as pain is directly limiting participation in daily life [29]. Pain interference has also been found to mediate the relationship between pain and depression in paediatric chronic pain [20]. It is associated both with higher levels of anxiety [30] and with higher child and parent pain catastrophizing [27,31]. While pain catastrophizing has utility in that specific interventions can be developed to target it, and pain intensity is useful for patient-centred quick assessments of pain, pain interference provides a broader picture of the impact of pain on a patient’s life.

For the current study, the following research aims and hypotheses were examined:

(1) Determine changes in pain catastrophizing, pain intensity, and pain interference associated with completing an intensive interdisciplinary pain treatment program. We hypothesized that each of these variables would be improved at program discharge and 3-month follow-up relative to baseline.
(2) (a) Determine baseline associations of post-program pain catastrophizing scores. We hypothesized that higher baseline anxiety will be associated with higher pain catastrophizing at discharge and 3-month follow-up. (b) Determine baseline associations of post-program pain intensity scores. We hypothesized that higher baseline parent catastrophizing will not be associated with pain intensity at discharge and 3-month follow-up. (c) Determine baseline associations of post-program pain interference scores. We hypothesized that higher baseline depression will be associated with higher pain interference at discharge and 3-month follow-up.

## 2. Materials and Methods

### 2.1. Study Context

The IIPT program is based out of a tertiary care health and rehabilitation facility for paediatric patients in Calgary, Canada. It is 3 weeks in duration, running for 6–7 h a day, 5 days a week. Days consist of group and individual physiotherapy, psychology, academic time, occupational therapy, recreational and art therapy, and a group for parents. They are discharged at the end of 3 weeks and receive five follow-up appointments, the first 2-weeks after discharge and the last 24-months later. The program aims to develop emotional regulation and coping skills, enhance non-pharmacologic pain management, improve physical functioning in patients, and strengthen adaptive parenting strategies, having similar clinical interventions and goals as other IIPT programs [11]. IIPT care is provided by physicians, nurses, psychologists, and physiotherapists. It shares staff with its host rehabilitation day hospital including recreation therapy and occupational therapy. Facility wide services at the tertiary care centre provide academic supports as well as art therapy. Most patients and families also attended the Comfort Ability program (www.thecomfortability.com, accessed on 12 July 2023) prior to entry into the IIPT [32]. It is a workshop on managing chronic pain facilitated by members of the IIPT psychology teams. Six patients who did not have access to Comfort Ability workshops before treatment were required to have attended psychology sessions focusing on similar topics as Comfort Ability, such as coping skills and chronic pain education.

### 2.2. Participants

Participants were 12–18 years old and attended the IIPT program between August 2016 and May 2022. There were forty-five youth in total, with six coming from outside Alberta, the province where the program is based. To be admitted, they must have tried 2–3 other evidence-based pain treatments that did not lead to achieving their functional goals and had to experience significant impairment in their daily function (i.e., poor sleep, school attendance/performance, physical function, self-care, mood, recreation, and/or social function). Patients and families were informed that the IIPT program encourages an active self-management approach. Patients were not admitted to the program if they had a brain injury or developmental delay that would prevent a pain self-management approach, a conversion disorder, and/or a psychiatric illness that required stabilization, such as an acute psychiatric crisis. All patients in the program were offered a chance to participate in the study.

### 2.3. Procedures

This annually renewed study was first approved by the University of Calgary’s Conjoint Health Research Ethics Board (REB14-0162 and REB20-1464) on 12 December 2014. It was conducted in accordance with the Declaration of Helsinki. Written informed consent was obtained from both youth and their participating parent. The IIPT program coordinator emailed the families about participating in research. The interested families were then sent informed consent forms, one for the parent and one for the youth over REDCap^®^. If they clicked “yes” to participating, then they were sent their baseline questionnaire within two weeks of starting IIPT. On the first day of IIPT, wet signatures from both parents and youth were obtained on the consent forms.

### 2.4. Measures

Self-report measures were administered at intake, discharge, and 3-month follow-up. Surveys were delivered electronically via REDCap^®^ [33].

#### 2.4.1. Demographics

Child age, gender, type of pain, and length of pain problems were collected, along with parent gender, marital status, household income, and ethnicity. Child age and gender were included in our analyses as a baseline factor.

#### 2.4.2. Pain Intensity

Patients reported pain intensity over the last 7 days using an 11-point Numeric Rating Scale (NRS), with 0 representing no pain and 10 representing the worst pain imaginable [34].

#### 2.4.3. Parent and Child Pain Catastrophizing

The Pain Catastrophizing Scale Parent (PSC-P) and Child Version (PSC-C) were used. They are strong predictors of functional disability in children and adolescents with chronic pain [35,36]. Both versions have three subscales: rumination, magnification, and helplessness distributed across 13 items ranked on a 5-point Likert scale, with a total score ranging from 0–52. The PCS-P has the same items as the PCS-C but uses a parental perspective instead of the first person (e.g., “When my child feels pain…” rather than “When I feel pain…”) before each of the items (e.g., “…it’s terrible and I can’t stand it anymore.”) to capture parent catastrophizing.

#### 2.4.4. Patient-Reported Outcomes Measurement Information System (PROMIS^®^): Pain Interference, Anxiety, and Depression

PROMIS^®^ measures multiple domains of wellbeing across healthy and disease specific populations using a collection of questionnaires [28,37]. It includes separate scales for pain interference, anxiety, and depressive symptoms in youth. The pain interference scale measures how pain interferes with physical, psychological, and social functioning using 8 items with 5-point Likert ratings. The anxiety scale contains three main subcategories that questions assess: hyperarousal, fear, and anxious misery (i.e. worry). This study used the short form, which contains 8 items with 5-point Likert ratings. The depression domain contains four main categories: mood, anhedonia, views of the self, and social cognition. Youth were administered the 8-item short form depression scale. Within the PROMIS^®^ system, score totals are converted to a T-score, whereby a T-score of 50 and standard deviation of 10 represents the general population average. Pain interference, anxiety, and depression each have their own T-score [37]. Each of these separate scales have been validated in a paediatric chronic pain population [38].

#### 2.4.5. Paediatric Health-Related Quality of Life (PedsQL^TM^)

The PedsQL^TM^ inventory measures health-related quality of life across four subscales: physical, emotional, social, and school [39]. It consists of 23 items ranked on a 5-point Likert scale. Items are linearly transformed to a 0–100 scale in each domain, whereby higher scores indicate a higher health-related quality of life.

### 2.5. Data Analysis 

This is a study of 45 participants who have completed the IIPT program. Data normality of demographic and baseline (intake) measures was tested using the Shapiro-Wilk test (*p* < 0.05). Continuous data was presented as mean ± standard deviation (SD) or median [interquartile range (IQR)] as appropriate. Regardless of the Shapiro-Wilk test result, demographic and baseline measures included in our linear mixed models were presented as mean ± SD along with median [IQR] (if appropriate) as these variables were mean centred for that analysis. Count data was presented as frequency (percentage).

For the purposes of this analysis, each participant presented with a cluster of three outcome measurements of interest, those being PCS-C, pain intensity, and pain interference collected at baseline, discharge, and 3-month follow-up. Intraclass coefficient correlations were explored for each outcome to assess this degree of clustering. Following the finding of high intraclass correlation coefficients (ICC) within our outcome variables (lowest ICC was 0.53 associated with pain interference), we used linear mixed models to provide an initial overview of how demographic and baseline measures are associated with PCS-C, pain intensity, and pain interference scores at discharge and 3-months following discharge from the IIPT program. A priori selected variables collected at baseline included in mixed model analysis were PCS-C, pain intensity, pain interference, depression, anxiety, PedsQL^TM^, PCS-P scores, age, and gender [13,14,40,41,42]. All a priori selected variables listed above except for demographic variables were captured at multiple time points. For this study however, we chose a narrow objective, which was to explore associations between baseline measures and outcomes captured at discharge/3-month follow-up. By limiting our analyses to baseline variables and outcomes at discharge/3-month follow-up, we were able to present initial associations that may be important to target if we wish to ensure good outcomes upon discharge that are sustained at follow-up from our IIPT program.

#### 2.5.1. Analysis Methods for Research Aim 1

Univariable linear mixed model analysis was used to explore changes over time in PCS-C, pain intensity, and pain interference scores from baseline through IIPT program discharge to 3-month follow-up. Separate linear mixed models were developed for each outcome measure with time included as the sole independent variable. For each model, time was coded as a three-level factor variable with each level representing one of our three time points of measurement. As such, two dummy variables representing time of measurement at discharge and 3-month follow-up were included in each model with measurements captured at baseline set as the reference category. Participants were included as a random effect to account for participant variation in baseline levels measures. The models were specified as:

Y_participant|time_ = (γ_00_ + μ_0participant_) + β_1_Time_time1_ + β_2_Time_time2_ + ε_participant|time_

where Y_time|participant_ represents a participant’s outcome score at baseline, discharge, or 3-month follow-up, γ_00_ is the fixed intercept representing the mean outcome value at baseline (time 0), μ_0participant_ is the random intercept associated with each participant, β_1_ is the fixed effect slope at discharge (time 1), β_2_ is the fixed effect slope at 3-month follow-up (time 2), and ε is the unexplained variance in the outcome measure not captured by the model.

#### 2.5.2. Analysis Methods for Research Aim 2

Multivariable linear mixed models for each a priori selected baseline variable were developed to explore their associations with post-IIPT program pain catastrophizing, pain intensity, and pain interference scores. Each specific model included the corresponding baseline values of the variable of interest at the individual level as an independent variable. Outcome data-points included in the models were restricted to discharge and 3-month follow-up given the inclusion of baseline values as fixed effect variables. Time of measurement was also included in the models as a two-level categorical variable on account of outcome measure data-point restriction to discharge and 3-month follow-up time points. Measurements captured at discharge were set as the time reference category. The models were specified as:

Y_participant|time_ = (γ_00_ + μ_0participant_) + β_1_BaselineVariable + β_2_Time_time2_ + ε_participant|time_

where Y_time|participant_ represents a participant’s outcome score at discharge or 3-month follow-up, γ_00_ is the fixed intercept representing the mean outcome value at discharge (time 1), μ_0participant_ is the random intercept associated with each participant, β_1_ is the fixed effect slope of the baseline variable of interest, β_2_ is the fixed effect slope at 3-month follow-up (time 2), and ε is the unexplained variance in the outcome measure not captured by the model. Gender, age, and interaction between time of measurement and baseline measure were considered for possible model inclusion. Log likelihood tests were used to determine if the inclusion of these variables improved model fit (*p* < 0.05) in relation to initial multivariable models that included fixed effects of baseline measure and time of measurement. Continuous baseline measures were mean centred while associations with PCS-C, pain interference, depression, anxiety, PedsQL^TM^, and PCS-P were presented per 10-unit change (instead of 1-unit change) to improve coefficient and accompanying 95% confidence interval (CI) presentation. The coefficients reported for these analyses are the mean response of the outcome variable averaged across discharge and 3-month follow-up, adjusted for time, and based on a participant presenting with a unit increase in the baseline variable from the mean [i.e., one year of age from the mean, male gender compared to female, one point on the NRS pain intensity scale from the mean (10-points for PCS-C, pain interference, depression, anxiety, PedsQL^TM^, and PCS-P from the mean)]. For the purposes of this study, *p* < 0.05 was considered significant and no correction was made to the level of statistical significance to account for multiple testing.

#### 2.5.3. Sensitivity Analysis

A sensitivity analysis to compare our available data results was completed using imputed data multivariable linear mixed models (see supplemental information). Little’s test was initially used to assess if data was missing completely at random (*p* < 0.05). We imputed 30 models of the dataset (representing the approximate highest degree of variable missingness) with 20 iterations (to ensure variable convergence) using multiple imputed chained equations (MICE) with predictive mean matching (pmm) and logistic regression. We did this imputation using our outcome variables, a priori identified baseline variables of interest, and demographic variables of age and gender, as well as a wider set of available baseline variables including length of time experiencing pain problems, current pain score, degree bothered by pain (NRS scale), fear of pain, functional disability score, adolescent sleep wake scale (total score), family assessment device score, parent fear of pain score, parent depression score, and parent anxiety score (22 variables total). We assumed data was missing at random. The number of potential available donor candidates to replace the missing data point was restricted to three to account for our small sample size. Imputed linear mixed models were constructed and presented to match those developed for our available data analysis. Available and imputed data results were presented as a fixed effect coefficient with 95% CI and *p*-values. Presented imputed coefficients represented the pooled estimates from individual linear mixed models completed on each of our 30 imputed datasets. Data were analysed using SPSS 25.0 (IBM, Armonk, NY, USA) and R Studio version 2022.2.3.492 with R statistical software version 4.2.1 (The R Project for Statistical Computing, Vienna, Austria), using packages lmerTest (version 3.1-3) and mice (version 3.15.0) for linear mixed model analysis and data imputation, respectively [43,44].

## 3. Results

Child (patient) and parent demographics are presented in Table 1. PCS-C, pain intensity, and pain interference presented with 28% (37 out of 135 possible data points), 29% (39 out of 135 data points), and 29% missing data, respectively. For baseline variables included for data imputation, missing data ranged from 8.9% (4 out of 45 possible baseline data points) to 22% (10 out of 45 possible baseline data points) for baseline family assessment device and child fear of pain scores, respectively. Little’s test (*p* < 0.001) showed that data was not missing completely at random. Baseline variable characteristics between participants with missing and non-missing outcome data at each time point were further explored in the Supplemental Information (Tables S1–S3).

### 3.1. Pain Catastrophizing, Pain Intensity, and Pain Interference Outcomes over Time

PCS-C scores were −5.7 (−8.5 to −3.0) and −7.2 (−10 to −4.3) points lower at discharge and 3-month follow-up compared to baseline (Table 2a). Pain intensity values at discharge and 3-month follow-up were not significantly different than baseline (Table 2a). Pain interference presented with reductions of −4.7 (−6.9 to −2.4) and −5.1 (−7.6 to −2.5) from baseline at discharge and 3-month follow-up, respectively (Table 2a). Imputed results were similar to available data findings (Table 2b).

### 3.2. Baseline Associations with Discharge/3-Month Follow-Up PCS-C Scores

Participants who had baseline PCS-C, pain interference, depression, and anxiety scores that were 10-points greater than the sample mean were associated with discharge/3-month follow-up averaged PCS-C scores that were, respectively, 9.9 (7.2 to 13), 14 (8.7 to 19), 8.5 (5.1 to 12), and 6.2 (3.0 to 9.3) points higher than a participant with mean baseline values (Table 3). Participants with baseline PedsQL^TM^ scores 10-points higher than the mean were associated with decreased averaged discharge/3-month follow-up PCS-C scores of −4.1 points (−6.3 to −1.9) (Table 3) compared to a participant with a mean baseline value. All associations remained significant after missing data imputation. Age and gender were not associated with averaged discharge/3-month follow-up PCS-C scores in either available or imputed data analysis (Table 3).

### 3.3. Baseline Associations with Discharge/3-Month Follow-Up Pain Intensity

Participants with baseline pain intensity scores one-point higher than the sample mean presented with an averaged discharge/3-month follow-up pain intensity score that was 0.75 (0.47 to 1.0) points higher than a participant with a mean baseline intensity score (Table 4). No other baseline measures were significantly associated with discharge/3-month follow-up averaged pain intensity for both available and imputed data analysis (Table 4).

### 3.4. Baseline Associations with Discharge/3-Month Follow-Up Pain Interference T-Scores

Participants with baseline PCS-C, pain interference, depression, and anxiety scores that were 10-points greater than the sample mean were associated with average discharge/3-month follow-up pain interference scores that were, respectively, 4.6 (2.3 to 6.9), 7.8 (4.2 to 11), 4.3 (1.8 to 6.7), and 2.6 (0.32 to 4.8) points higher compared to an individual with mean baseline values (Table 5). Individuals with baseline pain intensity scores one-point higher than the mean had averaged post-IIPT pain interference scores that were 2.0 (0.60 to 3.4) points higher than an individual with mean baseline values (Table 5). Participants with baseline PedsQL^TM^ scores 10-points higher than the average had averaged discharge/3-month follow-up pain interference scores that were −2.8 (−4.1 to −1.4) points lower compared to a participant with mean baseline values (Table 5). Only baseline pain interference remained significantly associated with averaged post-IIPT program pain interference scores (Table 5). Age and gender were not associated with averaged post-IIPT pain interference scores (Table 5).

## 4. Discussion

This study took baseline age, gender, PCS-C, pain intensity, pain interference, anxiety, depression, PedsQL^TM^, and PCS-P, and examined their associations with PCS-C, pain intensity, and pain interference at IIPT discharge and 3-month follow-up. PCS-P, age, and gender were not associated with any outcome.

As predicted, PCS-C scores showed a significant reduction at discharge and 3-month follow up compared to baseline. Previously established clinical reference points for PCS-C severity include low (<15), medium (15–25), and high (>25), with each reference point being associated with clinically significant differences in functional disability, depressive symptoms, and anxiety [36]. Our results indicate a shift from a high level of catastrophizing (mean: 27) to a more moderate level at IIPT discharge (21.3) and 3-month follow-up (19.8), suggesting clinically significant associations across multiple dimensions of wellness.

Contrary to our hypothesis, pain intensity scores showed no significant change over time compared to baseline values. This may be partly explained by emerging research showing that pain intensity can be a lagging indicator of treatment response, often following behind other psychosocial improvements [45]. This may also be explained by variation between IIPT programs. Some IIPT programs have mixed results with pain intensity outcomes, although many have reported reductions in pain intensity over time [6,7]. There is considerable heterogeneity in IIPT curricula—for example, some programs may have more psychoeducational workshops, while others may place more emphasis on physical therapy [46]. Previous research has called for more clarity into the specific components of individual IIPT programs, which could allow for better interpretation of results [11]. Importantly, reducing pain intensity scores is not the primary objective of many IIPT programs. Many patients with chronic pain have been trying to reduce or avoid pain for most of their disease course. This can lead to avoidance patterns that limit daily functioning and increase distress. In contrast, IIPT focuses on accepting and learning to function with pain as opposed to eliminating it [47]. It is possible that the IIPT program in this study places greater emphasis on restoring function, and less on pain symptoms, than other programs. This is reflected in the observed improvement in our study population’s pain interference scores, which is indicative of increased function and participation in daily activities, despite their unchanged pain intensity.

We found that baseline pain catastrophizing was associated with discharge/3-month pain interference. This is reflected in previous research showing that higher levels of pain catastrophizing predicted more pain interference with age as a mediator, where the predictive ability of pain catastrophizing was strongest in adolescence and grew weaker as patients aged into adulthood [31]. However, baseline pain interference was not associated with discharge/3-month pain catastrophizing. We found that baseline pain interference was not associated with discharge/3-month pain intensity, despite literature showing that pain interference mediates the relationship between pain intensity and functional disability in cross-sectional data [20]. Interestingly, the opposite relationship between these variables did show an association—baseline pain intensity was associated with discharge/3-month pain interference. This may be because pain intensity did not significantly differ from baseline in this cohort, and higher baseline intensity may interfere with overall program success and adherence. Finally, baseline pain interference was associated with discharge/3-month pain catastrophizing. This is similar to previous research showing a reciprocal relationship between pain interference and psychosocial challenges in an older adult population [48].

As predicted, higher baseline anxiety was associated with higher pain catastrophizing at discharge and follow-up, and higher baseline depression was associated with higher post-program pain interference. This is supported by a previous meta-analysis which found strong associations between anxiety, depression, and pain catastrophizing [26], and previous research which showed associations between depression, anxiety, and pain interference in paediatric populations experiencing non-chronic pain [49,50]. The association between anxiety and depression and pain outcomes reinforces the importance of psychosocial interventions in IIPT such as Cognitive Behavioural Therapy (CBT) and Acceptance and Commitment Therapy (ACT) [51,52]. However, high anxiety has been shown to negatively impact CBT treatments for paediatric chronic pain [53]. Therefore, pretreatment interventions that target anxiety may optimize psychosocial treatments during IIPT. Ongoing research into differences between outcomes for patients receiving pretreatment for anxiety and those receiving no pretreatment would provide useful information. Anxiety was not associated with pain intensity outcomes in our population, which counters previous findings from the literature [14].

Baseline PCS-P was not associated with pain intensity scores after treatment, which is consistent with our hypothesis and with previous findings [14]. Baseline PSC-P also was not associated with PSC-C or pain interference scores after treatment. These results contrast with extant literature demonstrating the relationship between baseline parent characteristics and child pain-related outcomes. Previous research demonstrated a correlation between PCS-P and PCS-C in outpatient paediatric pain clinics [36], and parent catastrophizing has been associated with fear avoidance in paediatric chronic pain patients [27]. It is likely that parental behaviour still has an influence on the psychosocial wellbeing of their child, but that this relationship is multifactorial and cannot be reduced to a direct relationship between PCS-P and our outcome variables in a chronic pain population with high levels of disability.

In the present study, baseline age and gender were not associated with PSC-C, pain intensity, or pain interference. The lack of association with gender may be explained by our small sample size. However, it is also possible that gender is not associated with IIPT outcomes for other reasons—previous research on the same cohort in a broader sample including multi-modal therapy found that gender was not associated with pain interference during treatment [54]. While a previous systematic review on children and adolescents (ages 5–18) showed that girls experience more chronic pain than boys [1], another systematic review on young adults (aged 15–34) showed an equal prevalence of chronic pain between sexes [55]. Additional research is therefore needed to clarify gender differences in adolescents with chronic pain. Moreover, greater endeavours should be made to include queer and transgender youth in pain research, as preliminary research indicates pain experiences may differ within this population [56]. In regards to age, our results are in contrast with extant literature showing that older age at IIPT entry is associated with higher pain intensity [14]. The lack of association with age may be due to the narrow age range of youth who underwent this IIPT program and, like gender, may be explained by the smaller sample size of this study (see Table 1). However, older age may merely be a marker for a combination of other factors related to age such as a longer course of pain, more treatment attempts, higher perceived ineffectiveness of existing pain therapies, and more exposure to a healthcare system that often stigmatizes chronic pain. These factors may decrease patient buy-in to IIPT programming, leading to decreased participation, less adherence to program recommendations, and reduced integration of IIPT concepts and practices into daily life. If patients had fewer of these negative experiences across their disease course, they may not have worse outcomes despite being older. Future research could explore the experiences of youth accessing healthcare for chronic pain, including associations between experiences of stigma, buy-in to treatment, and IIPT outcomes.

We found a significant association between baseline PedsQL^TM^ and pain catastrophizing at discharge and follow-up, consistent with a recent meta-analysis which found a strong association between pain catastrophizing and quality of life [26]. Baseline PedsQL^TM^ was also associated with pain interference at discharge and follow-up, demonstrating a similar association to one found in paediatric patients experiencing non-chronic pain [57]. However, PedsQL^TM^ was not associated with pain intensity outcomes. Interestingly, a previous trajectory analysis found that having a lower social function score (a subscale of the PedsQL^TM^) before treatment was associated with improved pain intensity outcomes afterwards [14]. The authors suggest that the relative improvement in wellbeing that comes with a transition to a more understanding social milieu may reduce perceptions of pain intensity. It is possible that there are significant associations between individual PedsQL^TM^ subscales and pain intensity that are obfuscated when the subscales are presented as a total score. Future research should investigate the associations between baseline PedsQL^TM^ subscales and pain intensity after treatment.

This study has several limitations. While the sample size was comparable to other studies on IIPT programs [58,59,60], it was restricted to 45 patients. This is partly due to the small number of patients admitted to the program each year, which approximates 6 to 8 youth. While this study generated meaningful data to direct future research, conclusions from this work must be interpreted with caution given the small sample size and lack of power analysis. Unfortunately, such a small sample size was the likely culprit that prevented more comprehensive mixed model analyses whereupon the associations between baseline variables and outcome measures could have been presented at each time point instead of mean aggregated across discharge and 3-month follow-up. Indeed, initial models incorporating a time interaction with baseline variables were explored but ultimately failed to converge. A smaller pool of patients to draw on for research is a challenge inherent to IIPT and could be addressed in the future by multi-site collaboration. Given the small sample size, associations between outcomes and gender may not have been identified, although it is also possible that there is no significant difference in outcomes between genders in this age range. Moreover, over 73% of the cohort’s parents identified as Caucasian, suggesting there is a need for more research into outcomes of chronic pain treatment for racialized individuals [61], as their experience of pain may be shaped by their racialization [62]. Another possibility for different outcomes stems from different pre-treatment experiences between patients. Most patients attended the Comfort Ability program prior to admission, which is an outpatient chronic pain group facilitated by a psychologist [11], while six patients had individual sessions with a psychologist instead. Differences in outcomes between these groups of patients would be a worthwhile future study with a larger sample size.

Post-treatment outcome data was missing in some patients. This was compensated by using imputation strategies to maximize data available for analysis. When analysing missingness, data can be categorized into three classes: either it can be missing completely at random, at random, or not at random. Little’s test did show that the data was not missing completely at random. However, such a test does not state whether data is missing at random or not at random. Unfortunately, to confirm whether data is missing at random or not missing at random, the very data that is missing is required. Many of our baseline variables did not show differences between participants with and without missing data (see supplemental information). However, all baseline variables had missing data themselves. It is possible that there were systematic reasons as to why a participant did not report a value (e.g., a poor outcome) as changes in the strength of association coefficients were noted after imputation compared to available data findings. MICE imputation assumes that the data was missing at random and results must be interpreted with caution if data missing not at random is suspected, such as with the difference between the strength of associations in our imputed and available data. We presented our available data models as our primary method of analysis, with those supplemented using imputation for sensitivity purposes. However, it should be noted that van Ginkel et al. present the argument that the use of multiple imputation, even under a missing not at random assumption, generates less biased results compared to listwise deletion [63].

Imputation increased the sample size after data collection. During data collection, phone calls and automated questionnaire reminders were sent out to increase participation. These strategies have been shown to reduce loss to follow up in youth populations [64]. Midway through data collection in October 2020, two new strategies were implemented by the researchers to improve response rates: providing gift cards for survey completion and increasing research team in-person interactions with patients during their IIPT treatment. These changes have led to a significant improvement in patient retention at follow-up. Finding ways to increase response rates is important, as patients lost to follow-up in psychiatric programs often respond less to treatment and have worse symptoms [65].

The generalizability of this study is also limited by the differences between IIPT programming, professional support, and duration. While IIPT programs share a standard definition of at least three professionals providing treatment in a day program setting with shared treatment goals for 2–6 weeks, there is considerable variation between individual programs [46]. There is a call for more clear descriptions of interventions found to be effective so that they may be replicated [66]. This has been done previously for this IIPT program in research that used input from patients, families, and other stakeholders to see how various program components reflected the program’s underlying values [11]. Future publications explaining the components of other IIPT programs could allow for a richer analysis of their outcomes and facilitate better program design. Additionally, problems with generalizability could be addressed by future research collaborations between IIPT programs sharing similar principles and curricula.

## 5. Conclusions

In summary, higher baseline PCS-C, pain interference, depression, and anxiety scores were associated with higher PCS-C discharge and follow-up. These baseline variables, along with higher baseline pain intensity, were associated with higher pain interference at discharge and follow-up. It is likely that optimization of anxiety and depression before starting IIPT would be associated with improved PCS-C and pain interference outcomes, supporting the use of pretreatment interventions. Additionally, participants presented with significantly reduced PCS-C and pain interference scores at IIPT discharge and 3-month follow-up compared to baseline values. Pain intensity showed no significant change over time at discharge or 3-month follow-up compared to baseline. Research into the perceptions of pain in patients with stable pain intensity scores despite improvement in PCS-C and pain interference following IIPT could provide insight into these mixed outcomes.

## Figures and Tables

**Table 1:** Complete case baseline child and parent demographics. Presented as mean ± standard deviation, median [interquartile range], or number (percentage within category). Age and pain intensity presented with non-normally distributed data. However, both were mean centred for linear mixed model analysis. Therefore, both mean ± standard deviation and median [interquartile range] are presented here.

**Child Demographics**	
Age (years)	16 ± 1.6; 17 [15 to 17]
Gender (female)	28 (74%)
Type of pain	
Abdominal	2 (7.1%)
Nerve (neuropathic)	7 (16%)
Headache	8 (18%)
Pelvic	0 (0%)
Musculoskeletal	6 (13%)
Other	5 (11%)
Length of pain problems (years)	3.3 [1.6 to 5.0]
PCS-C (total score)	28 ± 11
Pain intensity (NRS scale)	5.8 ± 1.9; 6.0 [5.0 to 7.0]
PROMIS^®^ pain interference (T-score)	65 ± 6
PROMIS^®^ depression score	60 ± 11
PROMIS^®^ anxiety score	59 ± 13
PedsQL^TM^ score	46 ± 18
**Parent demographics**	
Gender (female-identifying)	38 (88%)
Marital status	
Single	2 (5%)
Married	33 (79%)
Separated/Divorced	18 (17%)
Household income	
$0–29,999	2 (5%)
$30,000–59,999	3 (7%)
$60,000–89,999	6 (15%)
≥$90,000	24 (59%)
Do not wish to answer	6 (15%)
Ethnicity	
Chinese	2 (4%)
Latin American	1 (2%)
South Asian	1 (2%)
Caucasian	33 (73%)
Other	5 (11%)
Do not wish to answer	3 (7%)
PCS-P (total score)	22 ± 8

**Table 2:** (**a**). Available data from univariable linear mixed models exploring associations between baseline child pain catastrophizing scale score (PCS-C), pain intensity, and PROMIS^®^ pain interference scores with measures captured at discharge and 3-month follow-up. (**b**). Imputed data from univariable linear mixed models exploring associations between baseline child pain catastrophizing scale score (PCS-C), pain intensity, and PROMIS^®^ pain interference scores with measures captured at discharge and 3-month follow-up.

	**(a)**					
	**Available Data**					
	**^a^ PCS-C (^d^ N = 42) **		**^b^ Pain Intensity (^d^ N = 43) **		**^c^ PROMIS^®^ Pain Interference (^d^ N = 42) **	
**Time of Measurement**	**Coefficient Change from Baseline (95% CI)**	***p* Value **	**Coefficient Change from Baseline (95% CI)**	***p* Value **	**Coefficient Change from Baseline (95% CI)**	***p* Value **
Discharge	−5.7 (−8.5 to −3.0)	<0.001	0.46 (−0.27 to 1.2)	0.230	−4.7 (−6.9 to −2.4)	<0.001
3-month	−7.2 (−10 to −4.3)	<0.001	0.03 (−0.70 to 0.78)	0.930	−5.1 (−7.6 to −2.5)	<0.001
	**(b)**					
	**Imputed Data**					
	**^e^ PCS-C (N = 45) **		**^f^ Pain Intensity (N = 45) **		**^g^ PROMIS^®^ Pain Interference (N = 45) **	
**Time of Measurement**	**Coefficient Change from Baseline (95% CI)**	***p* Value **	**Coefficient Change from Baseline (95% CI)**	***p* Value **	**Coefficient Change from Baseline (95% CI)**	***p* Value **
Discharge	−5.1 (−8.7 to −1.4)	0.007	0.36 (−0.51 to 1.2)	0.414	−4.2 (−6.8 to −1.5)	0.002
3-month	−6.4 (−11 to −2.4)	0.002	0.08 (−0.70 to 0.86)	0.832	−4.5 (−7.7 to −1.4)	0.006

PCS-C, pain catastrophizing score (child); CI, confidence interval; Note for (**a**): ^a^ mean PCS-C baseline value of 27 (24 to 31); ^b^ mean pain intensity baseline value of 5.5 (4.8 to 6.2); ^c^ mean pain interference baseline value of 65 (63 to 68); ^d^ number of participants who presented with a minimum of one outcome measure at either baseline, discharge or 3-month follow-up for linear mixed model analysis. Note for (**b**): PROMIS^®^, patient-reported outcomes measurement information system; ^e^ mean PCS-C baseline value of 27 (22 to 31); ^f^ mean pain intensity baseline value of 5.6 (4.9 to 6.3); ^g^ mean pain interference baseline value of 65 (62 to 67).

**Table 3:** Available and imputed data linear mixed model analysis exploring fixed effect associations between child pain catastrophizing scale (PCS-C) scores captured at discharge and 3-months following discharge from an intensive interdisciplinary pain treatment (IIPT) program and baseline (intake) measures.

	^a^ Available Data		Imputed Case (N = 45)	
Baseline Measure	^b^ Fixed Effect Coefficient, β_1_ (95% CI)	*p* Value	^b^ Fixed Effect Coefficient, β_1_ (95% CI)	*p* Value
Baseline PCS-C score (per 10 points) mean available data: 27; mean imputed data: 29	9.9 (7.2 to 13)	<0.001	5.7 (2.3 to 9.0)	0.002
Baseline pain intensity (NRS scale) mean available data: 5.8; mean imputed data: 5.5	1.7 (−0.65 to 4.1)	0.166	0.89 (−1.1 to 2.9)	0.381
Baseline PROMIS^®^ pain interference T-score (per 10 points) mean available data: 64; mean imputed data: 65	14 (8.7 to 19)	<0.001	7.3 (0.83 to 14)	0.028
Baseline PROMIS^®^ depression score (per 10 points) mean available data: 60; mean imputed data: 60	8.5 (5.1 to 12)	<0.001	5.1 (2.0 to 8.2)	0.002
Baseline PROMIS^®^ anxiety score (per 10 points) mean available data: 59; mean imputed data: 60	6.2 (3.0 to 9.3)	<0.001	3.7 (0.78 to 6.7)	0.014
Baseline PedsQL^TM^ score (per 10 points) mean available data: 46; mean imputed data: 46	−4.1 (−6.3 to −1.9)	0.001	−2.4 (−4.4 to −0.45)	0.017
Baseline PCS-P score (per 10 points) mean available data: 22; mean imputed data 23	0.65 (−5.1 to 6.4)	0.825	1.2 (−3.2 to 5.6)	0.589
Age (years) mean available data: 16; mean imputed data: 16	0.35 (−2.5 to 3.2)	0.814	0.08 (−2.2 to 2.4)	0.942
Gender (reference group female)	1.7 (−9.0 to 12)	0.761	1.1 (−7.2 to 9.3)	0.794

N, number; CI, confidence interval; PCS-C, pain catastrophizing scale—child; NRS, numerical rating scale; PROMIS^®^, patient-reported outcomes measurement information system, PedsQL^TM^, pediatric quality of life inventory; PCS-P, pain catastrophizing scale—parent; ^a^ Available data varied between 33 and 35 participants for each linear mixed model dependent on missing data associated with each baseline measure; ^b^ Individual linear mixed models were developed for each baseline measure (adjusted for time). Fixed effect coefficients (β_1_) represent the mean response of PCS-C averaged across discharge and 3-month follow-up adjusted for time and based on a participant presenting with a unit increase in the baseline variable from the mean.

**Table 4:** Available and imputed data linear mixed model analysis exploring baseline fixed effect associations between pain intensity (NRS scale) captured at discharge and 3-months following discharge from an intensive interdisciplinary pain treatment (IIPT) program and baseline (intake) measures.

	^a^ Available Data		Imputed Case (N = 45)	
Baseline Measure	^b^ Fixed Effect Coefficient, β_1_ (95% CI)	*p* Value	^b^ Fixed Effect Coefficient, β_1_ (95% CI)	*p* Value
Baseline PCS-C score (per 10 points) mean available data: 27; mean imputed data: 29	0.15 (−0.46 to 0.76)	0.639	0.19 (−0.39 to 0.77)	0.515
Baseline pain intensity (NRS scale) mean available data: 5.8; mean imputed data: 5.5	0.75 (0.47 to 1.0)	<0.001	0.44 (0.04 to 0.85)	0.035
Baseline PROMIS^®^ pain interference T-score (per 10 points) mean available data: 64; mean imputed data: 65	0.78 (−0.20 to 1.8)	0.132	0.49 (−0.39 to 1.4)	0.270
Baseline PROMIS^®^ depression score (per 10 points) mean available data: 60; mean imputed data: 60	0.33 (−0.29 to 0.95)	0.304	0.33 (−0.25 to 0.91)	0.258
Baseline PROMIS^®^ anxiety score (per 10 points) mean available data: 59; mean imputed data: 60	0.04 (−0.47 to 0.57)	0.868	0.09 (−0.40 to 0.57)	0.714
Baseline PedsQL^TM^ score (per 10 points) mean available data: 46; mean imputed data: 46	−0.29 (−0.65 to 0.08)	0.133	−0.27 (−0.63 to 0.09)	0.136
Baseline PCS-P score (per 10 points) mean available data: 22; mean imputed data 23	0.23 (−0.74 to 1.2)	0.651	0.27 (−0.38 to 0.92)	0.413
Age (years) mean available data: 16; mean imputed data: 16	−0.20 (−0.64 to 0.24)	0.369	−0.18 (−0.51 to 0.15)	0.283
Gender (reference group female)	0.83 (−0.70 to 2.3)	0.298	0.82 (−0.42 to 2.1)	0.192

N, number; CI, confidence interval; PCS-C, pain catastrophizing scale—child; NRS, numerical rating scale; PROMIS^®^, patient-reported outcomes measurement information system, PedsQL^TM^, paediatric quality of life inventory; PCS-P, pain catastrophizing scale—parent; ^a^ Available data varied between 32 and 34 participants for each linear mixed model dependent on missing data associated with each baseline measure; ^b^ Individual linear mixed models were developed for each baseline measure (adjusted for time). Fixed effect coefficients (β_1_) represent the mean response of pain intensity averaged across discharge and 3-month follow-up adjusted for time and based on a participant presenting with a unit increase in the baseline variable from the mean.

**Table 5:** Available and imputed data linear mixed model analysis exploring baseline fixed effect associations between patient-reported outcomes measurement information system (PROMIS^®^) pain interference T-scores captured at discharge and 3-months following discharge from an intensive interdisciplinary pain treatment (IIPT) program and baseline (intake) measures.

	^a^ Available Data		Imputed Case	
Baseline Measure	^b^ Fixed Effect Coefficient, β_1_ (95% CI)	*p* Value	^b^ Fixed Effect Coefficient, β_1_ (95% CI)	*p* Value
Baseline PCS-C score (per 10 points) mean available data: 27; mean imputed data: 29	4.6 (2.3 to 6.9)	<0.001	2.5 (−0.03 to 5.1)	0.053
Baseline pain intensity (NRS scale) mean available data: 5.8; mean imputed data: 5.5	2.0 (0.60 to 3.4)	0.009	0.71 (−0.87 to 2.3)	0.364
Baseline PROMIS^®^ pain interference T-score (per 10 points) mean available data: 64; mean imputed data: 65	7.8 (4.2 to 11)	<0.001	5.7 (1.6 to 9.7)	0.009
Baseline PROMIS^®^ depression score (per 10 points) mean available data: 60; mean imputed data: 60	4.3 (1.8 to 6.7)	0.002	2.4 (−0.63 to 5.4)	0.114
Baseline PROMIS^®^ anxiety score (per 10 points) mean available data: 59; mean imputed data: 60	2.6 (0.32 to 4.8)	0.033	1.6 (−0.71 to 3.9)	0.166
Baseline PedsQL^TM^ score (per 10 points) mean available data: 46; mean imputed data: 46	−2.8 (−4.1 to −1.4)	<0.001	−1.5 (−3.3 to 0.28)	0.093
Baseline PCS-P score (per 10 points) mean available data: 22; mean imputed data 23	0.17 (−3.5 to 3.9)	0.929	−0.11 (−2.8 to 2.5)	0.930
Age (years) mean available data: 16; mean imputed data: 16	−0.25 (−2.1 to 1.6)	0.792	−0.21 (−1.8 to 1.3)	0.789
Gender (reference group female)	−0.47 (−7.3 to 6.3)	0.893	−0.41 (−6.3 to 5.5)	0.891

N, number; CI, confidence interval; PCS-C, pain catastrophizing scale—child; NRS, numerical rating scale; PROMIS^®^, patient-reported outcomes measurement information system, PedsQL^TM^, pediatric quality of life inventory; PCS-P, pain catastrophizing scale—parent; ^a^ Available data varied between 32 and 34 participants for each linear mixed model dependent on missing data associated with each baseline measure; ^b^ Individual linear mixed models were developed for each baseline measure (adjusted for time). Fixed effect coefficients (β_1_) represent the mean response of pain interference averaged across discharge and 3-month follow-up adjusted for time and based on a participant presenting with a unit increase in the baseline variable from the mean.

## Data Availability

Data available on request due to ethical and privacy restrictions. The data presented in this study are available on request from the corresponding author. The data are not publicly available due to patient privacy.

## Supplementary Materials

The following supporting information can be downloaded at: https://www.mdpi.com/article/10.3390/children10071229/s1, Table S1. Baseline characteristics in participants with available and missing discharge and 3-month pain catastrophizing scale—child (PCS-C) scores. Table S2. Baseline characteristics in participants with available and missing discharge and 3-month pain intensity (NRS scale) scores. Table S3. Baseline characteristics in participants with available and missing discharge and 3-month PROMIS^®^ pain interference scores. References [67,68] are cited in the supplementary materials.
