# Supplementary material for: Baseline Factors Associated with Pain Intensity, Pain Catastrophizing, and Pain Interference in Intensive Interdisciplinary Pain Treatment for Youth
Source: Children (Basel). 2023 Jul 15;10(7):1229. doi: 10.3390/children10071229 (PMC10378082; doi:10.3390/children10071229)
Supplement: Supplementary file 1 [file children-10-01229-s001.zip › children-2446211-supplementary.pdf]

Supplemental Information

Data imputation using multiple imputation by chained equations

Twenty-two variables were included in our data imputation procedure using multiple imputation by chained equations (MICE). These variables included our outcome variables of interest: PCS-C scores, pain intensity, and PROMIS® pain interference scores; *a priori* identified baseline variables of interest: PCS-C scores, pain intensity, pain interference, PedsQL™ quality of life score, PROMIS® depression scores, PROMIS® anxiety scores, and PCS-P scores; demographic variables: age and gender; additional collected variables: length of pain problems, current pain score, bothered by pain score, fear of pain score, functional disability score, adolescent sleep wake scale (total score), family assessment device score, parent PROMIS® depression score, and parent PROMIS® anxiety score. The number of total variables included (22) in our imputation procedure follows that suggested by [1].

Several variables in addition to our outcomes of PCS-C, pain intensity and pain interference were assessed at multiple time points. These included length of pain problems, current pain score, fear of pain score, functional disability score, PedsQL™, depression scores (child and parent), anxiety scores (child and parent), adolescent sleep wake score, and PCS-P. Our objective was to explore associations between baseline variables and outcome measures at discharge and 3-month follow-up. As such, only values collected at baseline except for our three outcome variables were included in the imputation procedure.

Missing data was 28% for PCS-C and 29% for pain intensity and pain interference. Child fear of pain presented with 22% missingness, the highest amongst all baseline variables considered for inclusion in imputation. These percentages of missingness were below a maximum suggested threshold of 40% for multiple imputation [2]. Baseline characteristics prior to imputation were explored between participants with missing outcome data (PCS-C, pain intensity and pain interference) at discharge and 3-month follow-up (Tables 1S-3S). No significance differences were found in child-related baseline characteristics between participants with or without missing outcomes. Caution must be noted that presented statistical tests were not powered to detect a specific effect, nor were corrections made for multiple comparisons.

Prior to running MICE, Little's test was performed to determine if the data was missing completely at random (MCAR). A P value < 0.001 suggested that the data was not MCAR. Multiple imputation by chained equations assumes that missing data is missing at random (MAR). We cannot confirm as to whether the data was MAR or missing not at random (MNAR) as the ability to distinguish between these two mechanisms requires the very data that is missing [2]. Assuming that our data was missing at random, 30 imputed datasets were generated, each with 20 iterations to approximately match the highest percentage of variable missingness and ensure appropriate data convergence, respectively.

1. van Buuren, S.; Boshuizen, H.C.; Knook, D.L. Multiple Imputation of Missing Blood Pressure Covariates in Survival Analysis. Statistics in Medicine 1999, 18, 681-694, doi:10.1002/(sici)1097-0258(19990330)18:6<681::aid-sim71>3.0.co;2-r.
2. Jakobsen, J.C.; Gluud, C.; Wetterslev J.; Winkel P. When and How Should Multiple Imputation be Used for Handling Missing Data in Randomised Clinical Trials – A Practical Guide with Flowcharts. BMN Medical Research Methodology **2017**, 17, 197, doi:10.1186/s-12874-017-0442-1.

Table S1. Baseline characteristics in participants with available and missing discharge and 3-month pain catastrophizing scale – child (PCS-C) scores.

[a]Baseline Measure	Discharge			3-month follow-up		
	Available (N = 33)	Missing (N = 12)	P value	Available (N = 29)	Missing (N = 16)	P value
Age (years)	17 [15 to 17] 0 missing	16 [15 to 17] 7 missing	0.675	16 [15 to 17] 4 missing	17 [14 to 17] 3 missing	0.592
Gender (female)	26/33 (79%) 0 missing	2/5 (40%) 7 missing	0.103	18/25 (72%) 4 missing	10/13 (77%) 3 missing	1.00
Length of pain problems (years)	3.0 [2.0 to 5.0] 2 missing	5.0 [1.5 to 11] 7 missing	0.657	3.5 [2.0 to 5.0] 5 missing	3.5 [1.3 to 8.5] 4 missing	0.811
NRS pain intensity score	5.7 ± 2.0 2 missing	6.2 ± 1.1 7 missing	0.596	6.0 ± 1.8 4 missing	5.2 ± 2.1 5 missing	0.213
NRS current pain score	6.0 [5.0 to 7.0] 2 missing	7.0 [5.0 to 9.0] 7 missing	0.371	6.0 [5.0 to 7.5] 4 missing	6.0 [5.0 to 7.0] 5 missing	0.753
Bothered by pain score (NRS scale)	6.1 ± 2.8 2 missing	6.6 ± 2.9 7 missing	0.679	6.5 ± 2.4 4 missing	5.2 ± 3.5 5 missing	0.188
Fear of pain score	52 ± 17 3 missing	63± 15 7 missing	0.169	51 ± 17 4 missing	58 ± 18 6 missing	0.305
Functional disability score	27 [20 to 35] 2 missing	26 [17 to 28] 7 missing	0.563	26 ± 11 4 missing	27 ± 13 5 missing	0.786
PCS-C (total score)	27 ± 11 2 missing	31 ± 11 7 missing	0.467	28 ± 11 4 missing	28 ± 12 5 missing	0.844
PedsQL™ quality of life (total score)	47 ± 19 2 missing	42 ± 12 7 missing	0.608	47 ± 18 4 missing	44 ± 19 5 missing	0.627
PROMIS® depression score	62 [55 to 68] 2 missing	65 [62 to 67] 7 missing	0.476	61 ± 11 4 missing	60 ± 11 5 missing	0.885
PROMIS® anxiety score	59 ± 13 2 missing	63 ± 14 7 missing	0.469	60 ± 13 4 missing	59 ± 13 5 missing	0.831
PROMIS® pain interference score	65 ± 6.9 2 missing	65 ± 2.8 7 missing	0.987	64 ± 7.1 4 missing	66 ± 4.7 5 missing	0.580
Adolescent sleep wake scale (total score)	3.3 ± 0.93 2 missing	3.8 ± 1.2 7 missing	0.250	3.2 ± 1.0 4 missing	3.7 ± 1.0 5 missing	0.222
Family assessment device score	36 ± 5.5 3 missing	40 ± 5.4 1 missing	0.024	38 ± 5.8 1 missing	35 ± 5.6 3 missing	0.171
PCS-P (total score)	23 ± 7.6 4 missing	19 ± 9.0 1 missing	0.092	23 ± 7.3 2 missing	21 ± 10 3 missing	0.626
Parent fear of pain score	34 ± 14 5 missing	25 ± 9.9 1 missing	0.061	30 ± 14 3 missing	34 ± 13 3 missing	0.335
Parent PROMIS® depression score	52 [45 to 58] 4 missing	46 [38 to 55] 2 missing	0.316	48 [38 to 57] 2 missing	55 [50 to 62] 4 missing	0.072
Parent PROMIS® anxiety score	54 [49 to 62] 4 missing	56 [43 to 63] 2 missing	0.862	54 ± 8.1 2 missing	58 ± 9.1 4 missing	0.187

PCS-C, pain catastrophizing scale – child; NRS, numerical rating scale; PedsQL™, pediatric quality of life inventory; PROMIS®, patient reported outcomes measurement; PCS-P, pain catastrophizing scale-parent

[a]Continuous baseline measures presented as mean ± standard deviation or median [interquartile range] after completion of a Shapiro-Wilk test ($P < 0.05$). Differences in baseline measures between participants with available and missing outcome measures were assessed using independent samples t-tests or Mann-Whitney U tests as appropriate. Differences in the proportion of females was assessed using a Fisher's Exact test. No correction to the significance level of 0.05 was made to account for multiple comparisons. The number of missing baseline measures associated with available and missing outcome measures is included.

Table S2. Baseline characteristics in participants with available and missing discharge and 3-month pain intensity (NRS scale) scores.

[a]Baseline Measure	Discharge			3-month follow-up		
	Available (N = 32)	Missing (N = 13)	P value	Available (N = 30)	Missing (N = 15)	P value
Age (years)	17 [15 to 17]<br>5 missing	16 [14 to 17]<br>2 missing	0.518	16 [15 to 17]<br>4 missing	17 [15 to 17]<br>3 missing	0.842
Gender (female)	19/27 (70%)<br>5 missing	9/11 (82%)<br>2 missing	0.690	20 (77%)<br>4 missing	8/12 (67%)<br>3 missing	0.694
Length of pain problems (years)	3.5 [2.0 to 5.0]<br>6 missing	3.5 [1.8 to 11]<br>3 missing	0.654	4.0 [2.0 to 5.0]<br>5 missing	2.0 [2.0 to 10.0]<br>4 missing	0.432
NRS pain intensity score	6.0 ± 1.7<br>5 missing	5.0 ± 2.3<br>4 missing	0.155	6.0 [4.8 to 8.0]<br>4 missing	6.0 [4.8 to 7.3]<br>5 missing	0.654
NRS current pain score	7.0 [5.0 to 8.0]<br>5 missing	5.0 [3.5 to 7.0]<br>4 missing	0.064	5.9 ± 2.3<br>4 missing	5.9 ± 2.7<br>5 missing	0.979
Bothered by pain score (NRS scale)	6.0 [5.0 to 9.0]<br>5 missing	5.0 [1.5 to 7.0]<br>4 missing	0.053	6.1 ± 2.5<br>5 missing	6.2 ± 3.5<br>4 missing	0.908
Fear of pain score	54 ± 19<br>5 missing	50 ± 12<br>5 missing	0.568	50 ± 16<br>5 missing	60 ± 18<br>5 missing	0.252
Functional disability score	28 ± 12<br>5 missing	22 ± 10<br>4 missing	0.233	26 ± 12<br>4 missing	28 ± 12<br>5 missing	0.679
PCS-C (total score)	29.6 ± 11.8<br>5 missing	22 ± 6.1<br>4 missing	0.027	27 ± 10<br>4 missing	30 ± 13<br>5 missing	0.531
PedsQL™ quality of life (total score)	45.1 ± 18.4<br>5 missing	48 ± 16<br>4 missing	0.656	48 ± 18<br>4 missing	40 ± 15<br>5 missing	0.252
PROMIS® depression score	62 ± 11<br>5 missing	57 ± 10<br>4 missing	0.290	62 [55 to 69]<br>4 missing	62 [55 to 68]<br>5 missing	0.860
PROMIS® anxiety score	61 ± 13<br>5 missing	55 ± 12<br>4 missing	0.250	59 ± 13<br>4 missing	60 ± 12<br>5 missing	0.832
PROMIS® pain interference score	65 ± 7.2<br>5 missing	64 ± 3.3<br>4 missing	0.563	64 ± 6.2<br>4 missing	66.6 ± 7.1<br>5 missing	0.275
Adolescent sleep wake scale (total score)	3.2 [2.4 to 4.2]<br>5 missing	3.5 [3.1 to 4.1]<br>4 missing	0.228	3.3 ± 0.99<br>4 missing	3.5 ± 1.0<br>5 missing	0.728
Family assessment device score	38 ± 6.0<br>2 missing	35 ± 5.0<br>2 missing	0.159	37 ± 5.8<br>2 missing	37.3 ± 5.9<br>2 missing	0.684
PCS-P (total score)	23 ± 7.3<br>3 missing	20 ± 10.2<br>2 missing	0.350	23 ± 7.2<br>3 missing	20.7 ± 10.0<br>2 missing	0.464
Parent fear of pain score	31 ± 14<br>4 missing	32 ± 14<br>2 missing	0.887	31 ± 13<br>4 missing	32.2 ±14.0<br>2 missing	0.791
Parent PROMIS® depression score	48 [38 to 53]<br>3 missing	58 [53 to 64]<br>3 missing	0.001	51 [38 to 57]<br>3 missing	50 [45 to 61]<br>3 missing	0.509
Parent PROMIS® anxiety score	53 ± 7.9<br>3 missing	60 ± 8.1<br>3 missing	0.017	54 ± 8.8<br>3 missing	56 ± 8.0<br>3 missing	0.487

PCS-C, pain catastrophizing scale – child; NRS, numerical rating scale; PedsQL™, pediatric quality of life inventory; PROMIS®, patient reported outcomes measurement; PCS-P, pain catastrophizing scale-parent

[a]Continuous baseline measures presented as mean ± standard deviation or median [interquartile range] after completion of a Shapiro-Wilk test ($P < 0.05$). Differences in baseline measures between participants with available and missing outcome measures were assessed using independent samples t-tests or Mann-Whitney U tests as appropriate. Differences in the proportion of females was assessed using a Fisher's Exact test. No correction to the significance level of 0.05 was made to account for multiple comparisons. The number of missing baseline measures associated with available and missing outcome measures is included.

Table S3. Baseline characteristics in participants with available and missing discharge and 3-month PROMIS® pain interference scores.

[a]Baseline Measure	Discharge			3-month follow-up		
	**Available (N = 33)**	**Missing (N = 12)**	**P value**	**Available (N = 27)**	**Missing (N = 18)**	**P value**
Age (years)	17 [15 to 17]<br>0 missing	16 [15 to 17]<br>7 missing	0.648	16 [15 to 17]<br>4 missing	17 [14 to 17]<br>3 missing	0.705
Gender (female)	26/33 (79%)<br>0 missing	2/5 (40%)<br>7 missing	0.103	16/23 (70%)<br>4 missing	12/15 (80%)<br>3 missing	0.709
Length of pain problems (years)	3.0 [2.0 to 5.0]<br>2 missing	5.0 [1.5 to 10.5]<br>7 missing	0.626	3.5 [2.0 to 5.0]<br>5 missing	3.5 [1.8 to 9.3]<br>4 missing	0.633
NRS pain intensity score	5.7 ± 2.0<br>2 missing	6.2 ± 1.1<br>7 missing	0.596	6.0 [5.0 to 8.0]<br>4 missing	6.0 [4.5 to 6.5]<br>5 missing	0.536
NRS current pain score	6.0 [5.0 to 7.0]<br>2 missing	6.0 [5.5 to 7.0]<br>7 missing	0.354	6.0 [5.0 to 8.0]<br>4 missing	6.0 [4.5 to 7.0]<br>5 missing	0.582
Bothered by pain score (NRS scale)	6.0 ± 2.8<br>2 missing	6.6 ± 2.9<br>7 missing	0.679	6.5 ± 2.4<br>4 missing	5.5 ± 3.3<br>5 missing	0.299
Fear of pain score	52 ± 17<br>3 missing	63 ± 15<br>7 missing	0.169	51 ± 17<br>4 missing	58 ± 17<br>6 missing	0.223
Functional disability score	27 [20 to 35]<br>2 missing	26 [17 to 28]<br>7 missing	0.536	26 ± 12<br>4 missing	27 ± 12<br>5 missing	0.829
PCS-C (total score)	27 ± 11<br>2 missing	31 ± 11<br>7 missing	0.467	28 ± 11<br>4 missing	28 ± 11<br>5 missing	0.889
PedsQL™ quality of life (total score)	47 ± 19<br>2 missing	42 ± 12<br>7 missing	0.608	48 ± 18<br>4 missing	43 ± 18<br>5 missing	0.469
PROMIS® depression score	62 [55 to 68]<br>2 missing	64 [57 to 73]<br>7 missing	0.450	60 ± 11<br>4 missing	61 ± 11<br>5 missing	0.949
PROMIS® anxiety score	59 ± 13<br>2 missing	63 ± 14<br>7 missing	0.469	59 ± 13<br>4 missing	60 ± 13<br>5 missing	0.935
PROMIS® pain interference score	65 ± 6.9<br>2 missing	65 ± 2.8<br>7 missing	0.987	64 ± 7.3<br>4 missing	66 ± 4.6<br>5 missing	0.567
Adolescent sleep wake scale (total score)	3.3 ± 0.94<br>2 missing	3.8 ± 1.2<br>7 missing	0.250	3.2 ± 1.0<br>4 missing	3.6 ± 0.94<br>5 missing	0.345
Family assessment device score	36 ± 5.5<br>3 missing	40 ± 5.4<br>1 missing	0.024	38 ± 6.0<br>1 missing	35 ± 5.2<br>3 missing	0.177
PCS-P (total score)	23 ± 7.6<br>4 missing	19 ± 9.0<br>1 missing	0.092	23 ± 7.4<br>2 missing	21 ± 9.5<br>3 missing	0.691
Parent fear of pain score	34 ± 14<br>5 missing	25 ± 9.9<br>1 missing	0.061	30 ± 14<br>3 missing	34 ± 13<br>3 missing	0.400
Parent PROMIS® depression score	52 [45 to 58]<br>4 missing	46 [38 to 55]<br>2 missing	0.299	48 [38 to 53]<br>2 missing	56 [50 to 62]<br>4 missing	0.011
Parent PROMIS® anxiety score	54 [49 to 62]<br>4 missing	59 [43 to 63]<br>2 missing	0.859	53 ± 7.8<br>2 missing	58 ± 8.8<br>4 missing	0.053

PCS-C, pain catastrophizing scale – child; NRS, numerical rating scale; PedsQL™, pediatric quality of life inventory; PROMIS®, patient reported outcomes measurement; PCS-P, pain catastrophizing scale-parent

[a]Continuous baseline measures presented as mean ± standard deviation or median [interquartile range] after completion of a Shapiro-Wilk test ($P < 0.05$). Differences in baseline measures between participants with available and missing outcome measures were assessed using independent samples t-tests or Mann-Whitney U tests as appropriate. Differences in the proportion of females was

assessed using a Fisher's Exact test. No correction to the significance level of 0.05 was made to account for multiple comparisons. The number of missing baseline measures associated with available and missing outcome measures is included.
